# The Role of Type 2 Innate Lymphoid Cells in Allergic Diseases

**DOI:** 10.3389/fimmu.2021.586078

**Published:** 2021-06-09

**Authors:** Haocheng Zheng, Yi Zhang, Jiachuang Pan, Nannan Liu, Yu Qin, Linghui Qiu, Min Liu, Tieshan Wang

**Affiliations:** ^1^ School of Traditional Chinese Medicine, Beijing University of Chinese Medicine, Beijing, China; ^2^ Beijing Research Institute of Chinese Medicine, Beijing University of Chinese Medicine, Beijing, China; ^3^ Journal Press of Global Traditional Chinese Medicine, Beijing, China

**Keywords:** innate lymphoid cells, allergy, ILC2, asthma, atopic dermatitis, food allergy, allergic rhinitis

## Abstract

Allergic diseases are significant diseases that affect many patients worldwide. In the past few decades, the incidence of allergic diseases has increased significantly due to environmental changes and social development, which has posed a substantial public health burden and even led to premature death. The understanding of the mechanism underlying allergic diseases has been substantially advanced, and the occurrence of allergic diseases and changes in the immune system state are known to be correlated. With the identification and in-depth understanding of innate lymphoid cells, researchers have gradually revealed that type 2 innate lymphoid cells (ILC2s) play important roles in many allergic diseases. However, our current studies of ILC2s are limited, and their status in allergic diseases remains unclear. This article provides an overview of the common phenotypes and activation pathways of ILC2s in different allergic diseases as well as potential research directions to improve the understanding of their roles in different allergic diseases and ultimately find new treatments for these diseases.

## Introduction

Allergic diseases, including asthma, atopic dermatitis (AD), food allergies, and allergic rhinitis (AR), have caused a substantial public health burden, reduced quality of life, and even led to premature death. In recent decades, with the rapid growth of the global economy, allergic diseases have become one of the most impactful diseases in society. The prevalence of allergic diseases has increased significantly in both developed and developing countries, but the increase is more evident in developed countries (1). The pathogenesis of allergic diseases has long been the focus of immunology research, and the occurrence of allergic diseases is closely related to the immune system. The pathophysiologies of allergic diseases are dominated by IgE-mediated inflammation and the type 2 immune response (2), and type 2 helper T cells (Th2 cells) and type 2 innate lymphoid cells (ILC2s) play roles in the development of the type 2 immune response by releasing cytokines such as IL-4, IL-5, IL-9, and IL-13 (3). In addition, regulatory T cells (Tregs) are very important for maintaining immune tolerance for mucosal barriers, dendritic cells (DCs) are related to the interaction between adaptive and innate immunity, and natural killer cells (NK cells), monocytes, and macrophages play major roles in the occurrence and development of allergic diseases (2).

Innate lymphoid cells (ILCs) are innate immune cells that are difficult to identify due to the lack of cell surface lineage markers. ILC subtypes correspond to T cell subtypes and can be divided into five types, namely, NK cells, ILC1s, ILC2s, ILC3s, and LTi. There is a mirrored correlation between ILCs and T cells. NK cells correspond to CD8+ T cells, and Th1, Th2, Th17 cells correspond to ILC1s, ILC2s, and ILC3s. The related ILC and T cell subgroups have similar functions and are subject to similar regulatory pathways (4). ILC2s, corresponding to Th2 cells in adaptive immunity, are highly involved in many diseases, such as allergic diseases and diabetes, and are specifically correlated with inflammation, metabolism, tissue repair, and nervous system regulation (5).

ILC2s are tissue-resident cells that are predominantly distributed in mucosal tissues such as lung, small intestine, skin, and adipose tissue. ILC2s play essential roles in allergic diseases and in the development of type 2 inflammation. Although ILC2s are present in low numbers in various, they are uniquely indispensable for a variety of allergic diseases because they rapidly enhance type 2 inflammation (3). Understanding the role of ILC2s in different allergic diseases is of great significance for better studying the relationship between allergic reactions and the immune system.

Neither human nor mouse ILC2s express lineage markers, but ILC2s in the normal physiological state express surface molecules such as CD45, CD90, C-kit, Thy-1, and MHC-II. ILC2s in the active state can express KLRG1 and ST2 at high levels according to their tissue location and activity status (4, 6). It is worth noting that ILC2s also have memory functions, as mice stimulated with TSLP or IL-33 showed an increased number of ILC2s for nearly 4 months and could react more quickly after the next antigen stimulation (7).

## Initiation of the ILC2 Response During Allergic Reactions

**Figure 1:**
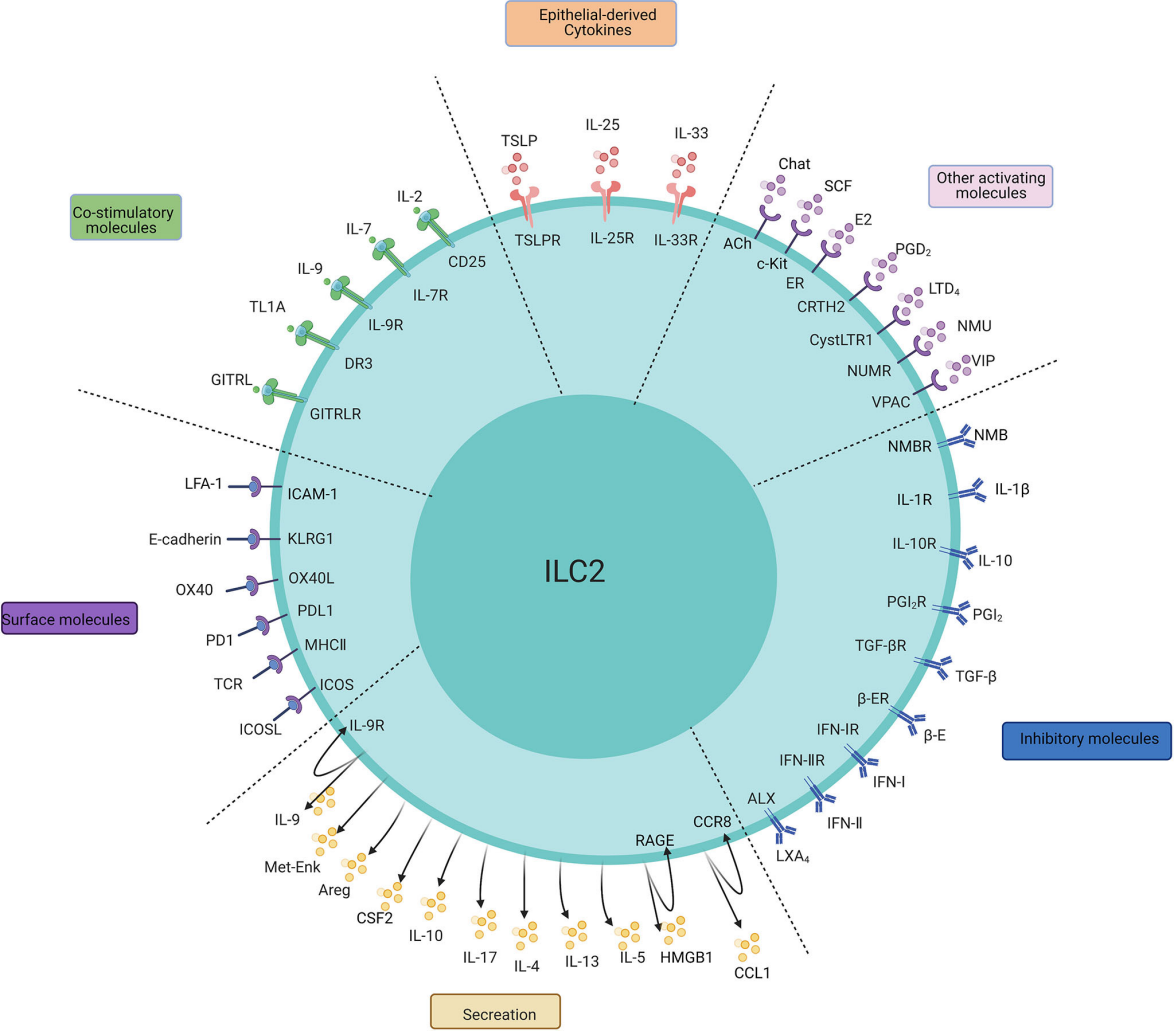
The main regulatory factors, surface molecules, and secretion of ILC2s. The PMIDs of relative articles: IL-1β: 32086822; IL-25:20023630,22079492; IL-33:21909091,23810766; TSLP:22425247,26129648, 29296700; IL-2:26595888; IL-7:24388011; IL-9:21983833; TL1A:24220298,24368564; GITRL: 29427641; E2:32648964; PGD2:24388011,31900341; LTD4:23688412; NMU:28869974; NMB: 32807943;SCF: 30617299;VIP:24037376; E-cadherin:24323357; OX40/OX40L:31470268; PD1:27749818; MHC-II:31320835; ICOS:25769613; IL-4:26883724; IL-9:21983833; IL-10:29196657; IL-17:29625134; Areg:26243875; Met-Enk:25533952;IL-10:29196657; TGF-β:25539814; IFN-I, IFN-II:26595888,26092469,26595887,26425820; PGI2, PGE2:26378386; β-adrenergic receptor:29496881; LXA4:23447017; CCL-1: 31537642; HMGB1: 32658914, 31937554.

Compared with the typical allergic response mediated by T cells and B cells, the ILC2-mediated response is faster and independent of antigen stimulation. ILC2s can further aggravate local inflammation and the immune response by releasing numerous cytokines that directly act on mucosal epithelia, blood vessels, and nerves or promote the responses of T cells and DCs. However, the activation of ILC2s is strongly correlated with the microenvironment and cell-to-cell signals. Cytokines, including IL-25, IL-33, TSLP, IL-2, IL-9, and IL-7, are necessary for the activation, proliferation, and maintenance of ILC2s (**Figure 1**) (6).

The major factors regulating ILC2s can be divided into three categories: IL-25, IL-33 and TSLP are major stimulating factors that are secreted by mainly epithelial cells to activate ILC2s; “IL-2, IL-4, IL-7, IL-9, TL1A, GITRL, etc. are survival factors, or so-called costimulators, that maintain the basic functionality of ILC2s; and other mediators that include lipid mediators (PGD2, LTD4), nervous peptides (neuromedin U (NMU), vasoactive intestinal peptide (VIP)), hormones (β-adrenaline), and the ILC2 inhibitory factor cluster (IL-10, TGF-b).

### Epithelial-Derived Cytokines

IL-25 and IL-33 are well-described signals that lead to a type 2 immune response and induce the differentiation of ILC2s. Both IL-25 and IL-33 can induce ILC2s to secrete IL-5 and IL-13 *via* the NF-κb or MAPK pathway (8, 9). Unlike the reliance of T cells on antigen peptide presentation for full activation, ILC2s can be activated directly by IL-33 in a state that has not been stimulated by antigens. However, mouse ILC2s hardly respond to IL-33 stimulation without costimulators (10). Studies have revealed that peroxisome proliferator-activated receptor-γ (PPARγ) is a member of the nuclear receptor superfamily, which regulates the transcription of target genes after ligand activation. The IL-33 stimulation of ILC2s results in high PPARγ expression on the cell surface, upregulation of the IL-33 receptor ST2, and promotion of the downstream release of IL-5 and IL-13. Therefore, a positive feedback loop exists between IL-33-ST2 signal transduction and PPARγ in the regulation of ILC2 activation (11). IL-25-induced ILC2s (KLRG1hiST2-ILC2s, also called inflammatory ILC2s or iILC2s) are functionally different from IL-33-induced ILC2s (KLRG1 intST2+ ILC2s, also called natural ILC2s or nILC2s) (12), their effects are not completely independent. IL-33 can promote the production of iILC2s by inducing the upregulation of tryptophan hydroxylase 1 (TPH1) expression (13). iILC2s can develop into nILC2-like cells and ILC3-like cells, and iILC2s can traffic from the intestine to the lung depending on the S1P protein (12, 14). Besides, IL-33 is more effective than IL-25 in airway diseases and AD (15). Other ILC2 subtypes, such as Thy-1+Sca-1+IL-18R+ST2-C-Kit- ILC2s, can produce IL-5 and IL-13 after *in vitro* stimulation to stimulate IgE production by B cells (16), and CD103+ ILC2s (dermis ILC2s) mainly reside in the skin (17). In addition, ILC2s selectively express different cytokine receptors in different tissues. ILC2s mainly express IL-33R in lung and adipose tissue, while ILC2s in the intestine mainly express IL-25R and skin ILC2s express IL-18R. In bone marrow, most ILC2s express both IL-33R and IL-25R, and a few ILC2s express IL-33R, IL-25R or IL-18R receptors (18).

### Survival and Activation Factors

Survival factors, or so-called costimulators, are necessary for maintaining the normal physiological function of ILC2s, and a lack of survival factor signals leads to their dysfunction. Survival factors include two main families, the common γ chain family and the TNF superfamily. The common γ chain family, including IL-2, IL-7, and IL-9, comprises regulatory factors necessary for ILC2 survival, development, and maintenance; the TNF superfamily, including TL1A and GITRL, is necessary for ILC2 proliferation and for their ability to release type 2 cytokines (6). The activation of ILC2s is closely related to the presence of IL-2 and IL-7, as mouse ILC2s can be activated only in the presence of IL-2 and IL-7 (19, 20), and human ILC2s can be activated only in the presence of IL-2 (21). In addition, IL-2 allows ILC2s to self-release IL-9 (22), and IL-9 signals are critical for the survival of ILC2s (23). IL-2-mediated ILC2 activation has been described in the lung, mesenteric lymph nodes (MLNs), spleen, and skin (17, 24, 25). It should be noted that ILC2s can still be activated in Rag-/- mice infected with *N. brasiliensis*, which suggests the diversity of IL-2 sources (26). Similarly, TL1A and IL-9 are essential for maintaining the activity of IL-25-induced and IL-33-induced ILC2s (22, 27, 28). However, in recent years, some researchers have reported that IL-7 may not be a necessary molecule for ILC2 development (29), and the importance of IL-7 in ILC2 development and activation needs to be further assessed. Stem cell factor (SCF) is also a key cytokine involved in the activation of ILC2s that binds to the receptor c-Kit on ILC2s and further amplifies the stimulatory effect of IL-25 on ILC2s to promote the release of downstream cytokines (30).

High-Mobility Group Box 1(HMGB1) is a late inflammatory mediator associated with sepsis, malignancy, and immune disease. HMGB1-dependent receptor for advanced glycation end products (RAGE) induces ILC2 expansion after hemorrhagic shock (HS), promotes ILC2 proliferation and promotes ILC2 survival by attenuating mitochondrial-mediated apoptosis. Type 2 cytokine secretion and eosinophil infiltration caused by ILC2 dilatation in the lung lead to lung injury after HS, and lack of HMGB1-RAGE signaling can reduce ILC2-induced type 2 inflammation (31). Besides, the activation of HMGB1-RAGE signaling pathway in ILC2 leads to an increase in the number of ILC2s secreting IL-13 and remodeling of the airway smooth muscle (ASM) (32).

In addition, many surface molecules and transcription factors are related to the activation of ILC2s. Airway epithelial cells infected by respiratory syncytial virus (RSV) can induce the production of uric acid, IL-33, TSLP and CCL2, the elevated level of uric acid further promotes the expression of innate cytokines, especially IL-1, by AECs and macrophages. Among these factors, CCL2 recruits monocytes, antigen-presenting cells (APCs) and Th2 cells to the lung, while IL-33, TSLP and IL-1β recruit and activate ILC2s (33). The CCL1/CCR8 autocrine signaling loop can regulate ILC2-mediated type 2 immunity and lead to infection resistance in worms, representing a newly discovered chemokine receptor-dependent mechanism (34). ICAM-1 is necessary for the development and function of ILC2s depend on ERK-GATA3 pathway. ICAM-1 deficiency leads to downregulation of the GATA3 protein, thereby resulting in ILC2 functional defects (35). ILC2s activated by IL- 33 significantly express LFA-1 and the intercellular adhesion molecule-1 (ICAM-1), the receptor of LFA-1. LFA-1 significantly promote the homing of ILC2s to the lung, and ICAM-1 deficiency significantly downregulates the levels of proinflammatory cytokines such as IL-5, IL-9, and IL-13 (36).

### Transcription Factors

RANK-L, the ligand of NF-κB, is mainly expressed in Th2 cells and CXCL16+ APCs in nasal polyps (NPs). The ligand of chemokine receptor 4 (CCR4) could recruits ILC2s and RANK-L+ cells into the NP and promotes the production of type 2 cytokines in ILC2s through a RANK-L-mediated pathway (37). In addition, c-Myc is a basic helix-loop-helix transcription factor mainly responded to IL-33, IL-25 and TSLP, which is closely related to the activation and pathogenicity of ILC2s *in vivo*. The activation of ILC2s results in the upregulation of c-Myc expression, which leads to proliferation and cytokine production of ILC2 (38). The AP-1 superfamily basic leucine zipper transcription factor, activating transcription factor-like (BATF) potentially regulates ILC2s. BATF defects cause selective damage to iILC2s in response to IL-25, are the early sources of IL-4 and IL-13 and serve as early guardians of mucosal barrier integrity (39). Runx proteins are a family of transcription factors necessary for many biological processes, and all Runx proteins require heterodimer formation with Subunit b of core binding factor (Cbfβ) (40). With the study of transcription factor Runx, the vital role of Runx in ILC2 proliferation and function has been recognized. The stimulation of Runx protein prevent ILC2 from overactivation which could inhibit the emergence of exhausted-like ILC2s during Allergic Inflammation (41).

### Lipoproteins

Lipoproteins, such as prostaglandins (PGs) and leukotrienes (LTs), the primary lipid mediators in the early stage of inflammation, are also active regulators of ILC2s. CRTH2 is used to identify human ILC2s, and PGD2, a CRTH2 ligand that is mainly expressed by mast cells, can promote the migration of ILC2s and the secretion of IL-13 (42, 43). Activated ILC2s also express high levels of LT receptors, and LTD4 is an effective stimulator of ILC2 activation and IL-5 and IL-13 production (44, 45). Although the secretion of IL-4 by ILC2s has rarely been reported, the stimulation of LTD4 allows ILC2s to produce IL-4 rather than IL-33 or IL-25 (44, 46). LTE4 LTC4 are also involved in the regulation of ILC2s, and LXB4 induces IL-13 production in mouse ILC2s (45, 47). Moreover, Group V phospholipase A2 (Pla2g5) is a lipid-generating enzyme that is required for the effects of macrophages on pulmonary inflammation. Macrophage-associated Pla2g5 plays an important role in type 2 immunity by regulating IL-33 induction and free fatty acid (FFA)-driven ILC2 activation (48).

### Neural Peptides and Hormones

ILC2s also have a strong relationship with the nervous system. VIP, a member of the neuropeptide secretin family expressed by intestinal cells, pancreatic neurons and suprachiasmatic nuclei of the brain, costimulates the VPAC2 receptor on ILC2s together with IL-7 and promotes the release of IL-5, inducing the generation and recruitment of eosinophils (49). NUM derived from mucosal neurons plays a vital role in ILC2 activation, proliferation, and secretion of type 2 cytokines. NMU stimulates the NMU receptor on ILC2s, which phosphorylates ERK1/2 and induces the activation of the intracellular Ca2^+^-calcineurin-NFAT cascade to induce the expression of downstream type 2 cytokines (50). Several studies have shown that type 2 cytokines and alarm factors can modulate neuropeptide release and further regulate the immune system. Interestingly, ILC2s are localized around several pulmonary neuroendocrine cells (PNECs) and regulated by PNECs through calcitonin, thereby stimulating the production of ILC2 factors. PNECs produce calcitonin gene-related peptide (CGRP) in combination with IL-7, IL-33 or IL-25, which can stimulate CGRP receptors on ILC2s and induce more IL-5 and IL-6 production (51). In addition, derivatives of testosterone and estradiol have regulatory effects on ILC2s (52, 53). Basophils can enhance the expression of the neuropeptide neuromedin B (NMB) receptor in mouse ILC2s, and NMB stimulation can inhibit ILC2-mediated type 2 inflammation (54). Compared with wild-type Esr1-/- mice, those stimulated by Alternaria extract (Alt Ext) exhibited fewer IL-33eGFP+ epithelial cells, reduced IL-33 release, reduced secretion of IL-5 and IL-13 and fewer bronchoalveolar lavage (BAL) eosinophils. It has been suggested that ER-α (Esr1) signaling increases the release of IL-33 and ILC2-mediated airway inflammation (55).

### Inhibitory Regulation of ILC2

ILC2s are downregulated by several inhibitory factors and regulatory T cells. IL-1β, derived from pulmonary macrophages, restricts type 2 inflammation and mucinous cell carcinoma after early rhinovirus infection by inhibiting the secretion of congenital cytokines such as IL-25 and IL-33 by epithelial cells, and this effect may be partially mediated by IL-17 (56). Moreover, IFN-I, IFN-II, and IL-27 can inhibit ILC2s, thereby limiting allergic airway inflammation in mice (57). Several lipid molecules were involved in the downregulation of ILC2, PGI2 and PGE2 are effective inhibitors of ILC2 activation (58), and LXA4 effectively inhibits cytokine production in ILC2s (59). Murine intestinal ILC2s express the β-adrenergic receptor, and the β-adrenergic pathway is a cell-intrinsic factor that negatively regulates ILC2 responses by inhibiting cell proliferation and effector functions (60).

Tregs play a key role in the inhibitory regulation of ILC2s, as an increase in Treg numbers leads to a decrease in the production of ILC2 cytokines, with IL-10 and TGF-β released by Tregs playing central roles in this process (61). Targeted deletion of RORα in mouse Tregs led to an exaggerated increase in the number of ILC2s (62). ICOS is an important pathway that is required for the proliferation, differentiation, and proinflammatory effects of ILC2s in various diseases. Tregs can also inhibit ILC2s through ICOS-ICOS-L, thereby controlling airway inflammation in mice, and the ICOS-ICOS-L pathway is integral to Treg-mediated ILC2 suppression (63–65). In contrast, ILC2-derived IL-4 could block Treg function to promote food allergies (66). Wu et al. also reported that PGD2 can activate ILC2s to release IL-5 through CRTH2, thereby inducing the production of CD4^+^CD25+IL5Rα+ Tregs (67). At the same time, ILC2s function in autocrine IL-9 signaling, and IL-9 can reduce Treg cell activation, leading to chronic arthritis. Treatment with IL-9 activated ILC2-dependent Tregs and effectively attenuated inflammation (68). In addition, Treg-dependent immunosuppression is correlated with ILC2 augmentation, and Tregs can induce tumor metabolic reprogramming *via* the STING/ILC2 axis (69). PD-1 is highly inducible in IL-33-activated ILC2s, PD-1 deficiency converts the ILC2 metabolic process into glycolysis, glutamine catabolism and methionine catabolism, enhances the activation and proliferation of ILC2s induced by IL-33, and regulates the production and survival of cytokines in aILC2s, including IL5, IL-13, IL-9 and CSF2 (70).

## Function of ILC2s in Allergic Reactions

**Figure 2:**
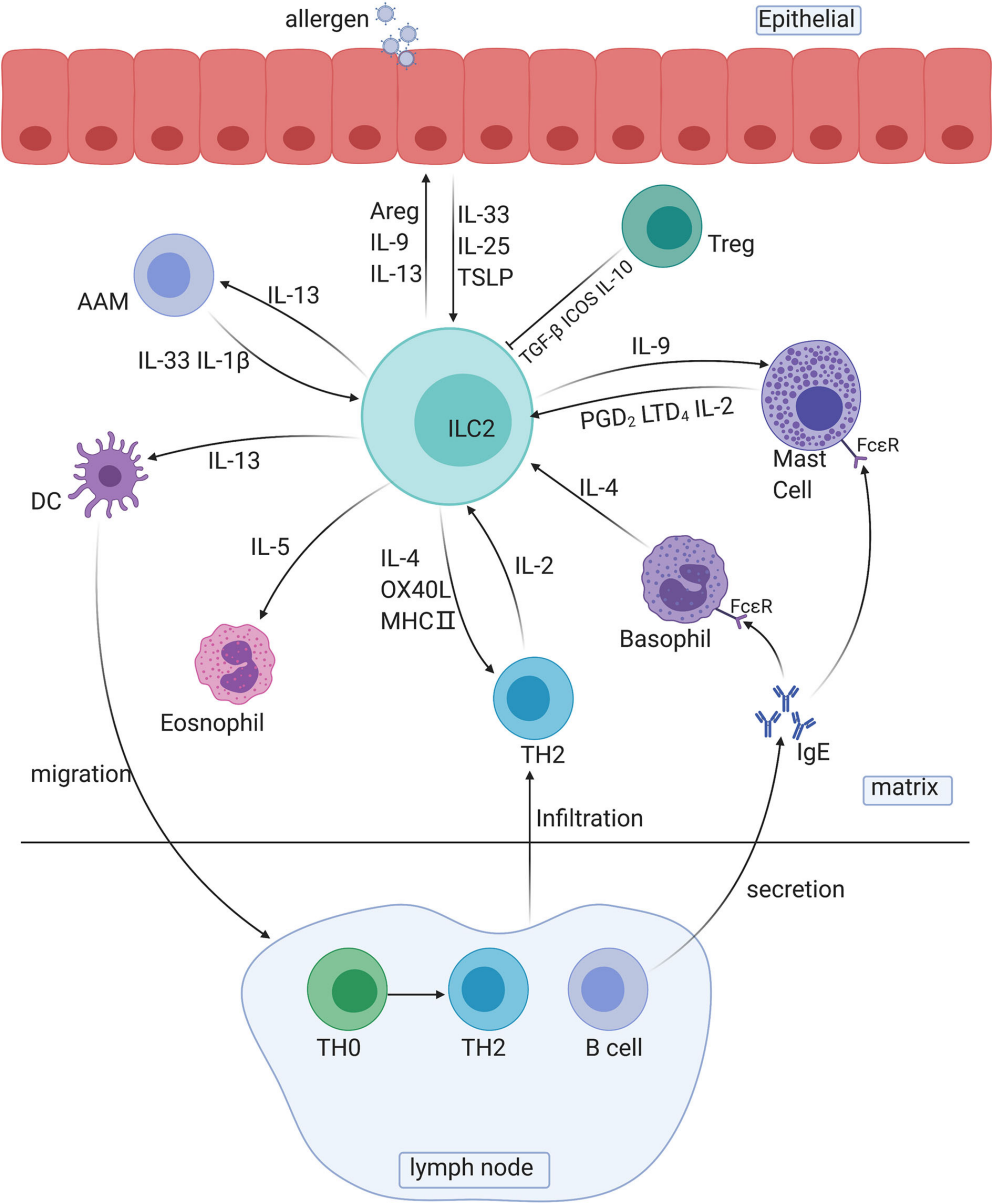
The roles of ILC2s in immune responses. AAM, alternative activated macrophage; DC, dendritic cell; TH cells, T helper cells; Areg, amphiregulin.

ILC2s respond quickly to allergic reactions mainly by secreting type 2 cytokines and other peptides, such as IL-4, IL-5, IL-13, IL-9, and amphiregulin (Areg), and by intercellular regulation of the cell-to-cell pathway. ILC2s are tissue-resident cells and can be quickly activated by several epithelial-derived cytokines, which allows ILC2s to release type 2 cytokines in the early stage of antigen sensitization (sensitization phase) (**Figure 2**).

Several cytokines secreted by ILC2s are involved in crosstalk with other immune cells: IL-13 is involved in the migration of activated DCs and activation of macrophages, and IL-5 is involved in the activation of eosinophils. For instance, the IL-33/ST2 axis is associated with bone marrow ILC2s and IL-5-dependent eosinophilia. Exposure to protease allergens increases the ST2 expression in bone marrow ILC2s, and the production of a substantial amount of IL-5 increases the number of eosinophils (71). IL-9 is involved in the activation of mast cells and ILC2s themselves. In addition, ILC2-derived IL-13 plays a decisive role in the migration of DCs to lymph nodes, which leads to the differentiation of Th2 cells and to the secretion of antibodies (72).

Crosstalk also exists between the two main innate immune components, complement components and ILC2s. C3a is a major driver of the ILC2-mediated inflammatory response to allergens and IL-33 and can increase and enhance the number and function of ILC2s induced by IL-33 and induce IL-13 and granulocyte-macrophage colony-stimulating factor (GMCSF) to inhibit IL-10 production in ILC2s. For ILC-T cell crosstalk, ILC2s exert antigen-presenting effects through C3a signaling. At the same time, C3a promotes the activation of MHC-II-dependent T cells, thus promoting the differentiation and proliferation of Th2 cells and further enhancing Th2 immunity (73).

Additionally, Madouri F et al. found that mice lacking PKC-θ had reduced ILC2 numbers, Th2 cell numbers, and AHR. Adoptive transfer of ILC2s restored eosinophil influx and IL-4, IL-5 and IL-13 production in the lung tissue as well as TH2 cell activation, demonstrating the vital role of PKC in the crosstalk between ILC2s and Th2 cells (74). ILC2s and Th2 cells, the leading promoters of the type two immune response in natural and adaptive immunity, are reciprocal activators during allergy initiation. Pelly found that ILC2-derived IL-4, which is induced by LTD4, is necessary for Th2 cell differentiation, and the lack of ILC2-derived IL-4 can lead to Th2 cell dysfunction (46). On the other hand, Oliphant reported that ILC2-derived IL-2, rather than IL-4, is essential for Th2 maintenance (24). In the recall phase, T cell infiltration conversely promotes the activity of ILC2s *via* the release of IL-2 and IL-4 and *via* B cells to promote the development of allergic reactions. Activated B cells release numerous IgE antibodies and bind to FCϵR on the surface of mast cells and basophils to enhance allergic responses. PAG1, a transmembrane connexin that affects the signal transduction of T and B cell receptors, can promote airway epithelial activation, ILC2 expansion and TH2 differentiation and aggravate the severity of asthma (75).

ILC2s also express several surface markers, such as ICOS ligand, MHC-II, and OX40L, which have broad effects on TH2 cells, B cells, and Tregs. ICOS belongs to the CD28 superfamily, and both human and mouse ILC2s express ICOS and its ligand (ICOSL), which can regulate the function of effector T cells (63). ILC2s play a role in APCs by upregulating MHC-II, regulating the Th1/Th2 balance, and transforming adaptive immunity into a Th2-type response (76). Lung ILC2s may regulate the proliferation and activation of CD4 T cells induced by RSV through the OX40/OX40L interaction (77). Death receptor 3 (DR3) is involved in inducing naïve and activated ILC2s to produce type 2 cytokines. The latest research shows that IL-33 can induce fat retention and the expression of DR3 in human peripheral blood ILC2s and participate in canonical and/or noncanonical NF-κB pathways to improve glucose tolerance and regulate the metabolic homeostasis of adipose tissue; thus, IL-33 can be used to treat patients with type 2 diabetes (T2DM) (78). Tumor necrosis factor-like protein 1A (TL1A) and the receptor DR3 costimulate T cells and ILC2s and play roles in a variety of autoimmune and allergic diseases. For example, the expression of RORα in resident T cells in the skin can upregulate the expression of DR3, promote T cell function, and inhibit allergic dermatitis. Therefore, the RoRα and TL1a-DR3 pathways can be used as therapeutic targets for allergic dermatitis (62). ILC2s can interact with Tregs through ICOS/ICOS L and IL-4, thereby inhibiting Treg activity. Tregs can downregulate ILC2 function *via* TGF-b and IL-10 (65, 66).

ILC2s can destroy the integrity of the mucosa by secreting IL-13, thereby making it easier for allergens to enter the stroma through the epithelium (79). However, most ILC2s are associated with mucosal repair. Mucosal barrier dysfunction can lead to the release of epithelial alarm molecules, including IL-33, IL-25, and TSLP, thereby activating ILC2s. In contrast, ILC2s can promote epithelial cell repair by producing Areg. IL-33 can be used to treat intestinal inflammation through the Areg-EGFR pathway (80). The secretion of IL-4 and IL-13 by ILC2s is crucial for the formation of AAMs, which are involved in the repair of epithelial cells (81). In addition, IL-9-mediated ILC2 survival is necessary for maintaining tissue integrity and lung function (23). ILC2s can release IL-9 to prevent epithelial cell apoptosis in mice with sepsis (82). Simultaneously, some researchers have shown that IL-9 secreted by ILC2s has a specific protective effect on inflammation. IL-9 treatment can promote ILC2-dependent Treg activation and effectively reduce inflammation, which may explain the unique role of IL-9 in arthritis (68).

## ILC2s in Allergic Asthma

Chronic airway inflammation commonly presents clinically as AR or chronic rhinosinusitis (CRS) in the upper airway and as asthma in the lower airway (83). A total of 19-38% of AR patients have concomitant asthma, and a much higher proportion of asthma patients have concomitant AR (84). This indicates that these diseases share some pathophysiological and immunological pathways, in which the type 2 immune response and IgE-mediated inflammation play a vital role. ILC2s, as newly identified participants in the innate immune system, have a wide range of effects on these diseases.

The prevalence of asthma has increased by almost 30% in the past two decades. Over 30 million patients worldwide suffer from it each year, which poses a heavy burden on public health services (85). Asthma is a chronic inflammatory respiratory disease characterized by AHR, type 2 inflammation, and airway remodeling. Classically, Th2 cells play an important role in the development of asthma by releasing type 2 cytokines.

### Role of ILC2s in Asthma

The numbers of both blood ILC2s and bronchoalveolar lavage fluid (BALF) ILC2s were shown to be increased in asthmatic mice, which contributed to the development of experimental asthma *via* the rapid secretion of IL-5 and IL-13 to aggravate lung eosinophilia and mucus production and exacerbate AHR (86, 87). In the house dust mite (HDM) mouse model, ILC2-deficient mice showed decreased IgE levels (87). In patients with severe asthma patients, the levels of ILC2s in the blood and sputum are increased significantly (88). These results suggest that ILC2s are highly involved in asthma.

ILC2s are the predominant population of ILCs in the lung in the steady state but still represent only a small group of immune cells in the lung (89). The persistence of the mouse asthma model has been proven to be dependent on ILC2s rather than antigen-specific T cells (20, 90). In a mouse model of asthma, IL-25-induced ILC2s and IL-33-induced ILC2s contributed 50~80% of the total IL5 and IL-13 production in the lung (91), although stimulation with IL-25 or IL-33 separately did not cause excessive lung reactions (92). It should be noted that different airway allergens may play different roles in the activation of ILC2s. Helfrich found that different airway models and mouse strains exhibited vastly different ILC2 numbers and distribution patterns in the lung and MLNs, suggesting the necessity of using multiple mouse strains to study ILC2 function in asthma (93).

In asthma, after activation by epithelial-derived alarm cytokines, ILC2s promote pulmonary inflammation by secreting type 2 cytokines, including IL-4, IL-5, and IL-13, to enhance the contraction of smooth muscle, secretion of mucus, and infiltration of inflammatory cells (94). IL-2 was found to effectively aggravate the inflammatory response in the lungs. The loss of IL-2 was shown to decrease ILC2 function, thereby reducing the lung inflammation caused by asthma in IL-2-deficient mice (25). Basophil-derived IL-4 and T cell-derived IL-2 are necessary for establishing rapid allergic reactions mediated by ILC2s (25, 95). In addition, CD127 (IL-7 receptor) is generally considered to be a marker of ILC2s, and some researchers have found that ILC2s can be activated only in the presence of IL-7. However, CD127low ILC2s have also been identified in asthma patients and mice, and it is worth noting that CD127 expression was shown to be decreased after IL-25 stimulation (12). The dependence of ILC2s on IL-7 in asthma and the regulatory mechanism of IL-7 on ILC2 function remain unclear and need further study.

In Rag-/- and WT mice, inhalation of papain caused mucus production, which was alleviated by IL2rg knockout, and mucus production resumed after the adoptive transfer of ILC2s, which indicated the critical role of ILC2s in inducing mucus secretion (96). The enhancement and activation of eosinophils are decisive for increasing airway mucus. LTD4 and papain can potentiate eosinophil and ILC2 proliferation in Rag-/- mice, and a lack of ILC2s leads to a decrease in the number of eosinophils (20, 44). Some scholars further verified that IL-5 and IL-13 released by ILC2s are the key cytokines responsible for eosinophil induction. Lack of ILC2-derived IL-5 and IL-13 leads to a decrease in the number of eosinophils (97, 98). These results also corresponded to the early increase in eosinophil numbers found in asthma models and suggest that ILC2s can enhance the activation of eosinophils in the early stage of asthma to produce mucus. At the same time, IL-13 produced by ILC2s could directly affect epithelial and smooth muscle cells, thereby inducing the production of AHR, mucus secretion, and airway remodeling in patients with allergic asthma. Researchers found that H3N1-infected Rag-/- mice could still develop AHR, but the AHR response disappeared after the depletion of ILC2s with Thy1.2 (19). ILC2s can produce IL-13 to stimulate the differentiation of macrophages into AAMs and promote AAM accumulation, which can lead to airway inflammation and airway remodeling (99, 100).

ILC2s also affect the differentiation of Th2 cells and IgE production by B cells, which is the key process in the development of asthma. ILC2s stimulate the migration of DCs into MLNs by secreting IL-13 to further promote the differentiation of T cells and B cells (72), and loss of ILC2s results in Th2 cell functional deficiency (72, 88). ILC2s can also stimulate the function of B cells through ICOS ligands, which are necessary to enhance airway inflammation and AHR (63). Interestingly, transcriptomic and epigenetic studies of human and mouse ILC2s showed that these cells were positively correlated with genes known to be responsible for asthma susceptibility (including *RORA*, *Smad3*, *GATA3*, *IL13*, *il18r1*, and *il1rl1*), suggesting that ILC2s are downstream regulators of some susceptibility genes in asthma development (101).

Although ILC2s are thought to induce inflammation in patients with asthma, researchers found that they also protect against inflammation by releasing IL-9. ILC2s could prevent the apoptosis of epithelial cells in subjects with sepsis through the secretion of IL-9, but whether ILC2-derived IL-9 plays an essential role in asthma remains unclear (82). Some researchers discovered a group of new ILC2 subsets that have the ability to produce IL-10 under IL-2 stimulation to further reduce inflammation in the lungs. It is worth noting that IL-2 is also an effective cytokine for inducing the production of IL-9 in ILC2s (22), suggesting that the IL-2-ILC2 axis functions in tissue repair.

Previous research has described the crosstalk between the nervous system and asthma (102). Sui et al. found that PNECs could regulate ILC2s, thus enhancing the allergic reaction in asthmatic mice by releasing neuropeptides (51). Wallrapp et al. have shown that the NUM protein can also enhance allergic inflammation in the lungs by activating ILC2s (103). In addition, Lauriane found that LC2s express the α7-nicotinic acetylcholine receptor (α7nAChR), which is thought to play an anti-inflammatory role in several inflammatory diseases. The α7nAChR agonist could decrease ILC2-dependent airway hyperreactivity by downregulating the transcription of GATA-3 and NF-κB (104). Given the strong correlation between the nervous system and asthma, the regulatory mechanism of ILC2s and the nervous system in patients with asthma is worthy of investigation.

Steroid therapy is a conventional treatment for asthma, but patients often develop resistance, thus leading to refractory asthma (105). Research shows that patients with severe refractory asthma have significantly higher airway ILC2 levels than those without asthma. Systemic steroids, rather than inhaled steroids, could relieve symptoms and reduce the number of ILC2s (106). In addition, the steroid resistance caused by ILC2s is dependent on TSLP activation, and TSLP-induced steroid resistance can be reversed by MEK and STAT5 inhibitors (107, 108). These findings provide insights into treatments targeting the TSLP pathway in steroid-resistant patients. Additionally, Ma Suli found that the levels of the membrane molecule OX40L were significantly increased in patients with steroid resistance, and OX40L was shown to be highly expressed in ILC2s (109).

### ILC2 Recruitment in the Lung

Unlike Th2 cells, which reside in lymphatic tissue and mostly migrate to tissues only after activation, ILC2s have been described as tissue-resident cells. Upon the occurrence of inflammation, ILC2s exert local immune effects after stimulation by the corresponding cytokines. However, ILC2s have the potential to translocate from one tissue to another. Huang et al. found that ILC2s could migrate from the intestine through lymphatic channels dependent on the S1P protein, thereby aggravating asthma (14). In addition, LFA-1 can significantly control the recruitment of ILC2s to the lungs, which indicates that it is also involved in the migration of ILC2s to the lungs (36). BATF plays a key role in the migration of IL-25-induced ILC2s, and migratory ILC2s are an important source of IL-4 and IL-13 in the early response stage (39).

## ILC2s in Allergic Rhinitis

### Role of ILC2s in Allergic Rhinitis

AR is characterized by IgE-mediated inflammation, which results in increased numbers of Th2 cells and type 2 cytokines in the nasal mucosa (110). Some studies found that after intranasal allergen challenge in cats, the numbers of CRTH2^+^ ILC2s in the peripheral blood were increased significantly (111). Peng et al. identified the distribution of ILC2s on the nasal mucosa membrane through immunohistochemistry and found that the number of ILC2s in the nasal mucosa was positively correlated with the AR clinical visual analog scale (VAS) score (112). In contrast, two articles reported that the numbers of peripheral blood ILC2s were not significantly altered in patients with AR and in artemisinin-induced mice (86, 113). Additionally, Kato assessed the role of ILC2s in AR by using a ragweed-induced mouse model and found that, compared with that in the PBS group, sneezing either disappeared or became significantly less frequent over time in Rag2-/- mice, suggesting that T cells, not ILC2s, are central to the development of AR (114). All of these findings indicate that ILC2s are far less important in AR than in asthma, and more reliable evidence is needed to determine how ILC2s are involved in the AR response.

Some studies have focused on the initiation pathway of ILC2s in AR. Allogeneic and autologous Myeloid dendritic cells (MDCs) were shown to activate ILC2s in patients with AR to produce type 2 cytokines and increase GATA-3 signal transduction and transcription factor activation. MDCs promoted ILC2 function in AR patients through the IL-33/ST2 pathway, and pDC activation inhibited ILC2 function through IL-6 (112). There are several reports of multiple lipid receptors, including CysL1R (LTD4 ligand) and PGD2, that are upregulated in AR patients. Although LTD4 was shown to activate IL-4 production in ILC2s, it should be noted that the levels of IL-4 in the nasal secretions of AR patients were not significantly changed (47). In addition, PGD2 has a chemotactic effect on CRTH2+ ILC2s in the blood of patients with AR and may directly regulate the migration of ILC2s into tissues (113). Whether lipid molecules regulate AR through ILC2s needs to be further investigated. Ozone has been shown to aggravate AR and asthma by inducing the release of IL-5 and IL-13 from ILC2s (115).

It is worth noting that the number of ILC2s in patients with HDM-induced AR was significantly higher than that in those with mugwort-AR (116). The peripheral blood ILC2 content was considerably higher in human pediatric patients with HDM-AR than in those without HDM-AR (117). The possible mechanism underlying this difference is that HDM is more immunogenic than plant-derived allergens, and its sensitization mechanism is presumably different from that of plant-based allergens, such as wormwood pollen (118).

## ILC2s in Allergic Dermatitis

### Role of ILC2s in Allergic Dermatitis

AD is a multifactorial complex inflammatory skin disease that involves barrier dysfunction and severe itching caused by chronic eczema (119). According to a report published in 2016, the 12-month adult prevalence of AD was 4.9% in the US, 3.5% in Canada, and 4.4% in the EU, with values for individual countries ranging from 2.2% for Germany to 8.1% for Italy. Among those countries, the US had the highest incidence rate of severe AD. Dermatitis was reportedly one of the most significant causes of increased years lived with disability (YLD) values and the most prevalent skin condition in China in 2017, with 1.39 million cases being reported, which was increased by 5.05% compared with that in 1990 (1.32 million) (120).

The pathological process of AD is characterized by a type 2 immune response and a rapid increase in IgE, which is accompanied by eosinophil infiltration, mast cell activation and involvement of other related lymphocytes in the production of typical type 2 cytokines, such as IL-4, IL-5 and IL-13. AD always represents the first step in “atopic march”, which leads to other allergic diseases, including asthma and AR. The pathological process of AD is divided into 3 stages: the nonlesional skin stage, acute lesional stage, and chronic lesional stage. The early stage is initiated by innate immunity, in which ILC2s play an important role. The acute stage is characterized by a noticeable type 2 immune response and inflammation, and the chronic stage mainly involves Th1 inflammation and the infiltration of various inflammatory cells (121, 122).

Overexpression of IL-33, IL-25, and TSLP has been observed in patients with AD (122). However, it should be noted that IL-33 may play a more prominent role in the activation of ILC2s in patients with AD, as ILC2s isolated from lesions predominantly responded to IL-33 rather than IL-25 and TSLP. At the same time, IL-33 induced ILC2 migration and cytokine production, whereas only TSLP at a high concentration combined with IL-25 induced these phenomena (122–124). IL-33-induced AD in mice relies on ILC2s, which are the primary mechanism responsible for the development of human AD (124). A clinical trial on etokimab, an IL-33 antibody, for the treatment of AD also revealed the vital role of IL-33 in mediating human AD (125). It has also been indicated that nILC2s but not iILC2s mainly inhabit healthy human skin (122). An experiment utilizing transgenic mice with a high level of skin-specific IL-33 indicated that the mice exhibited spontaneous dermatitis with AD-like inflammation (126). In contrast, the depletion of ILC2s *via* the transplantation of bone marrow from RORα-deficient mice into IL33tg mice (a type of transgenic mouse overexpressing IL-33) reduced IL-33-induced AD-like inflammation (127). A study performed in 2019 proved that eliminating T and B cells in IL33tg mice did not reduce symptoms, such as skin inflammation and increased numbers of ILC2s and type 2 cytokines (124).

Of note, CD103+ ILC2s, unique to the skin, were described as dermal ILC2s (dILC2s), which are distinct from other ILC2 subgroups (17). This group of ILC2s was shown to promote the activation of mast cells, the recruitment of eosinophils and the production of IL-5 *via* IL-2 treatment (17). The dermal ILC2-mediated expansion of DCs involves the expression of CCL17, which is vital for the TH2 memory cell response (128, 129). This illustrates that ILC2s are critical for orchestrating an efficient localized memory TH2 cell response in collaboration with tissue-resident DCs. Simultaneously, these results demonstrate the presence of various phenotypes of ILC2s in the skin, but their contributions to AD need to be further assessed.

High numbers of basophils were detected in subjects with AD-like inflammation, accompanied by ILC2 proliferation and upregulation of IL-5 and IL-4 (124). Basophils were also shown to be involved in the exacerbation of AD-like inflammation and activation of ILC2s *via* IL-4. After activation by basophils, ILC2s not only clearly accumulate but also enhance the expression of CCL11, IL-5, IL-9 and IL-13, which further leads to the accumulation of eosinophils and promotes other inflammatory responses in subjects with allergic diseases (124, 130). Cysteinyl leukotrienes (CysLTs), including LTC4, LTD4 and LTE4, are also effective molecules for moderating ILC2s and promoting the process of AD. Among the three LTs, LTE4 may have the greatest effect on ILC2 proliferation and induce the production of IL-4, IL-5 and IL-13 (44, 131). Moreover, ILC2s can regulate T cells through MHC-II, with only 3% of ILC2s in the skin and 50% of ILC2s in the skin-draining lymph nodes of mice expressing MHC-II (129, 132).

It should be noted that in the BALB/C mouse AD model induced by MC903, IL-25 had a greater effect on activating ILC2s than IL-33, but it was less important than TSLP. In contrast, TLSP was more vital than IL-33 and IL-25 in the C578BL/6 murine model (122). AD can be simulated in a variety of mouse models, which have skin symptoms and pathological changes that are similar to those of humans. Although considerable differences exist among each mouse AD model, human skin-derived ILC2s primarily respond to IL-33 (122, 123).

### Outside-in Hypothesis—Local Inflammation Induced by Skin Disorders

Filaggrin (FLG), a filament-associated protein that binds to keratin fibers in epithelial cells, is highly associated with AD development. Seventy-three percent of AD patients carry at least 1 FLG null mutation, and FLG-deficient mice present symptoms of spontaneous skin lesions (133). Gene-edited mice were generated to simulate FLG deficiency in humans, and the frequency of ILC2s was increased in the lesions of FLG-deficient mice (134, 135). In addition to FLG, the proteins filaggrin-2, hornerin, and the cornified envelope precursor SPRR3 are also linked to the onset of AD, but the mechanism needs to be further studied (136–138). The tight junction proteins between cells and the stratum corneum are skin barriers that prevent the invasion of microorganisms. The absence of epidermal keratins, such as FLG, promotes the binding of microorganisms to the skin and further leads to an inflammatory reaction, which is a major mechanism of AD development. More than 90% of AD patients exhibit lesions with *Staphylococcus aureus*, while only 5% of healthy people have *Staphylococcus aureus* (139). After microorganisms break through the skin barrier, the innate immune system initiates an initial response first, which leads to the activation of adaptive immunity. Additionally, the immune statuses of AD patients with lesional and nonlesional skin are significantly different. Thus, scientists have proposed that the pathogenesis of AD is related to epidermal dysfunction caused by genetic mutations, microbial infections, and other factors that are critical drivers of AD. ILC2s, as the predominant producer of type 2 cytokines in the early stage that have MHC-II and Toll-like receptor (TLR) activity, are broadly involved in the response to microorganisms. Damage to epithelial cells leads to the production of alarm factors to activate ILC2s.

### Immune Disorder Hypothesis—Immune-Mediated Skin Disorders

The incidence of AD is related to not only genetic factors and microorganisms but also endogenous factors, such as diet, pressure and living conditions. Internal factors must exist that regulate the pathogenesis of AD. Some studies found that over 50% of patients with severe AD had asthma, AR, or food allergies, suggesting the importance of immune system-mediated skin dysfunction in the pathogenesis of AD (140). IL-4, IL-13, IL-22, and IL-13 can reduce the protein expression of FLG and activate the release of bradykinin (141, 142), and the effectiveness of anti-IL-4 and anti-IL-13 in AD treatment shows the importance of the type 2 immune response in humans with AD (143). Moreover, immunosuppressive therapies and immunotherapies, such as steroids, were shown to inhibit the epidermal responses of clinical AD patients (144), suggesting that epidermal dysfunction induced by immune system abnormalities is another mechanism leading to the development of AD. Although ILC2s can repair epidermal damage through Areg, various phenomena suggest that ILC2s are likely key factors that aggravate skin inflammation in AD patients. However, the specific role and mechanism of ILC2s in epidermal repair and injury still need further exploration.

## ILC2s in Food Allergies

### Role of ILC2s in Food Allergies

Food allergies are incurable, and allergen-specific IgE antibodies are usually produced, which mediates hypersensitivity. At present, the development of food allergy sensitivity remains poorly understood, but IL-9 is known to induce the release of mucosal tissue mast cells (MMC9), basophils and Th2 cells, and Treg cells play a significant role in this process (145). The intestinal mucosa contains a large number of ILC2s, which promote allergic reactions by releasing IL-4, IL-5, IL-9 and IL-13. Scholars have also discovered the important role of ILC2s in the pathogenesis of food allergies (146). In addition, because the ingredients in food are mostly nonself-antigens, the tolerance established by gastrointestinal lymphoid cells such as Tregs is essential, and the reduction or loss of Tregs is a crucial mechanism underlying food allergies.

Food allergies can be divided into two types: IgE-mediated and non-IgE-mediated. In general, DCs and other APCs present antigens to Treg cells to build immune tolerance (147). However, in some cases, the Treg pathway is inhibited or transformed into Th2 cell-activated and IgE-driven immune responses. Sribava found that the ILC2 content in the small intestine was increased in mice with IgE-mediated food allergies (148), and the content of ILC2s was decreased in the small intestine after *GATA-1* activity was reduced, indicating the roles of ILC2s and *GATA-1* in IgE-mediated food allergies (146). In addition, ILC2s can inhibit the formation of Treg-mediated tolerance by releasing IL-4 and further aggravate food allergies (66).

Although food allergies are typically considered to be IgE-mediated allergic inflammatory diseases, the association between the IgE concentration and the allergic reaction severity is weak (145). IgE may not be the only factor determining the severity of the disease, as scientists have discovered the importance of IL-25 and IL-33 in food allergies, and IL-33 has been shown to stimulate Th2 cells (149), mast cells and ILC2s to promote the type 2 immune response and IgE release (150, 151). In addition, IL-33 was shown to be necessary for the development of a peanut allergy mouse model (152). ILC2s can also release IL-5 and IL-13 under the stimulation of TH2 cells and IL-25, which is vital for the development of eosinophilic inflammation (153).

Animal food allergy models rely on IL-4-mediated immune responses, and IL-4 can decrease Treg-mediated tolerance, which is a critical mechanism of food allergies. It is worth noting that the IL-4 in subjects with food allergies is primarily derived from CD4^+^ T cells rather than ILC2s, and IL-4 released by ILC2s has no discernible effect on food allergies (154). In ILC-deficient mice, eosinophilic inflammation was significantly weakened, but the Th2 activation, IgE production, and allergic reactions were not significantly changed (154). Correspondingly, Th2 cells rather than ILC2s seemingly play a decisive role in food allergy production. The depletion of CD4 cells or Th2 cells made it difficult for mice to develop an allergic response (153). However, some researchers have reported that ILC2-derived IL-4 is necessary for the development of peanut allergies (154). In addition, the importance of IL-9 in food allergies has been gradually elucidated. MMC9 induction is key to the development of IgE-mediated food allergy sensitivity, which is the main factor mediating intestinal hypertrophy (155). The production and development of MMC9 require stimulation with exogenous IL-9 signals, while ILC2s have the ability to secrete IL-9 (156). It is not clear whether ILC2-derived IL-9 and MMC9 interact with each other and affect food allergies.

### Multiple Routes of Food Sensitization

Epidemiological investigations have revealed significant correlations between food allergies and other allergic diseases, as approximately 50% of patients with food allergies suffer from AD (157). Some patients with food allergies present symptoms after the first contact with a certain kind of food, and peanut allergies and the use of skin preparations containing peanut oil are significantly correlated (158). This suggests that sensitization is established *via* routes other than oral administration. Earlier studies showed that oral, sublingual, nasal contact, and skin contact antigens could induce food allergies and be reproduced by oral administration (159). Skin exposure to allergens promoted the sensitization of mice and activation of Th2 cells to aggravate peanut allergies (149). Subsequently, researchers found that IL-33, but not IL-25 or TSLP, led to significant changes in skin exposed to peanuts, which was sufficient to activate Th2 cells. This indicates that further studying the contribution and mechanism of ILC2 in multiple food sensitization routes is worthwhile.

## Conclusion

Although ILCs have been known to exist for only approximately one decade, their role in the immune system is increasingly valued. As mirrors of TH2 cells, ILC2s play a pivotal role in the type 2 response, especially in allergic diseases. As indicated in the current research, ILC2s seemingly play a more important role in asthma and AD than in AR and food allergies, and there are few studies on ILC2s in AR and food allergies. Therefore, substantial evidence needs to be collected to assess the role of ILC2s more clearly.

Compared with TH2 cells, ILC2s are much more involved in the early allergy stage, especially in the induction of eosinophilia. ILC2s can be activated without antigen presentation and thus play a unique role in allergic diseases. ILC2s respond more quickly and have a better recognition ability than other cells. IL-33 induces ILC2s to produce 80% of the IL-5 and IL-13 found in the lungs. ILC2s, to some extent, make up for some of the regulatory functions of TH2 cells in the immune response. In addition, the normal function of ILC2s requires support from other cells *via* the secretion of IL-2, IL-7, and IL-9. In short, the ILC and adaptive immune systems play complementary roles in the development of allergic diseases.

Current research on ILC2 subtypes remains incomplete. Although some scientists have confirmed the existence of two major ILC2 subtypes, nILC2s and iILC2s, whether more functional subtypes exist and the roles of ILC2s in allergies still need to be determined. In addition, allergic disease treatments based on the ILC2 axis offers a new approach to the discovery of allergy drugs. It is imperative to better understand the basic functions, distributions, and classifications of ILC2s and to further explore the relationships between ILC2s and other immune cells, which will enable us to comprehensively understand allergic diseases.

## Author Contributions

Our article takes the opinions of allergists in different fields in order to provide an accurate approach to the current progress and research direction of several allergic diseases. HZ is responsible for asthma and food allergies parts, JP is responsible for AD part, and YZ is responsible for AR part. Besides, the else contribution is as follows: Review and Editing: HZ, YQ. Language polish: LQ. Visualization: YZ. Funding acquisition: ML. Supervision: TW. All authors contributed to the article and approved the submitted version.

## Funding

This research was funded by National Natural Science Foundation of China (No.81774375).

## Conflict of Interest

The authors declare that the research was conducted in the absence of any commercial or financial relationships that could be construed as a potential conflict of interest.
